# Nicotine e-cigarettes in Peru: analysis of law 32159 against international evidence and gaps for adolescent protection

**DOI:** 10.17843/rpmesp.2026.431.15979

**Published:** 2026-03-31

**Authors:** Jesus Chirinos-Caceres, Eva Chanamé-Ampuero, Abel Limache-García, Alfonso Zavaleta-Martínez-Vargas

**Affiliations:** 1 School of Public Health, Administration and Social Sciences, Faculty of Public Health and Administration, Universidad Peruana Cayetano Heredia, Lima, Peru.; 2 Information and Education Center for the Prevention of Drug Abuse (CEDRO), Lima, Peru.; 3 Department of Nursing, Faculty of Nursing, Universidad Peruana Cayetano Heredia, Lima, Peru.; 4 Department of Cellular and Molecular Sciences, Faculty of Sciences and Engineering, Universidad Peruana Cayetano Heredia, Lima, Peru.; 5 Faculty of Medicine, Universidad San Ignacio de Loyola, Lima, Peru.

**Keywords:** Electronic Cigarettes, Electronic Nicotine Delivery Systems, Public Health, Smoking Prevention, Tobacco Control, Peru

## Abstract

The article analyzes the regulation of nicotine electronic cigarettes (EC) in Peru, with an emphasis on Law 32159 and its capacity to protect the health of adolescents in light of international evidence. Although they are often perceived as less harmful than combustible cigarettes, studies link their use to cardiovascular and respiratory risks, as well as a higher probability of transitioning to combustible tobacco use in adolescents. The evolution of the Peruvian regulatory framework for tobacco control is reviewed, and it is recognized that Law 32159 constitutes an advance by explicitly incorporating electronic nicotine delivery systems within a comprehensive regulatory approach. In this sense, the law addresses 100% smoke- and vape-free environments, the prohibition of advertising, promotion, and sponsorship, health warnings, marketing restrictions, multisectoral oversight, and incorporates Art. 5.3 of the Framework Convention on Tobacco Control regarding industry interference in control policies for the consumption of tobacco products, nicotine, and substitutes for both. However, gaps persist between the law and its implementation: insufficient definitions of flavorings, lower requirements for health warnings, and limited capacity to control indirect and digital advertising in a context of informal markets and online trade. The absence of fiscal measures weakens public policy. In conclusion, the impact of the law will depend on precise regulations, effective oversight, and complementary instruments such as price and tax policies.

## INTRODUCTION

In 2018, the National Academies of Sciences, Engineering, and Medicine (NASEM) published the public health consequences of electronic cigarette (EC) use ^1^. Previously, on May 10, 2016, the Food and Drug Administration (FDA) issued a rule ^2^ to extend regulation to all tobacco products, including electronic cigarettes, as they meet the legal definition of a tobacco product. This “Deeming Rule” ^2^ allows the FDA to regulate the manufacturing, distribution, and marketing of tobacco products such as e-cigarettes and includes automatic provisions such as restrictions on sales to youth. Although various forms of electronic nicotine ^3^ provided by batteries have existed for more than a decade, their popularity, especially among youth, has increased in the last five years ^4^. Unlike conventional cigarettes (CC), electronic cigarettes do not burn and do not contain most of the estimated 7,000 chemical constituents present in tobacco smoke. Therefore, electronic cigarettes are believed to be safer than CC; however, they do produce exposure to nicotine and a variety of other potentially harmful components ^1^. Recent evidence based on an umbrella review that integrated 69 systematic reviews suggests that the use of electronic nicotine delivery systems is associated with higher cardiovascular and respiratory risks, as well as mental health issues and substance abuse, with particular concern for adolescents. Among the reported findings are increases in heart rate and blood pressure, as well as decreased lung function and a higher probability of asthma in users ^5^. Harm also occurs if youth begin their tobacco use with electronic cigarettes and then transition to combustible ones, or if adult CC smokers add e-cigarettes to complement their smoking habit instead of quitting combustible tobacco cigarettes completely ^1^. The objective of this article is to perform a critical analysis of the Peruvian regulations applicable to nicotine EC, including its historical evolution and Law 32159 ^6^, contrasting its regulatory provisions with selected evidence and relevant standards to identify specific gaps and implementation challenges, without developing an extensive review of their health effects.

## SCOPE

**Figure 1:**
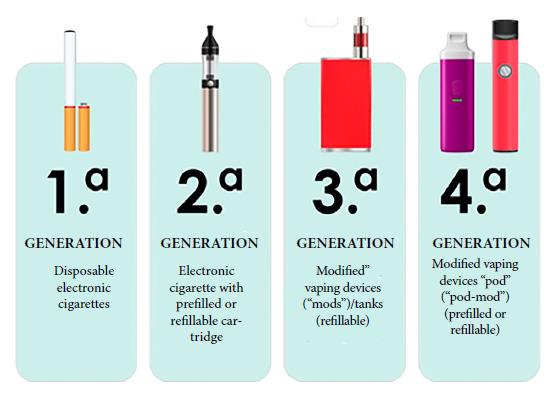
Electronic Nicotine Delivery Systems (ENDS) are classified by generation.

Source: CDC. (2019) e-cigarette, or vaping, products and dictionary. URL: https://www.cdc.gov/tobacco/basic_information/e-cigarettes/pdfs/ecigarette-or-vaping-products-visual-dictionary-508.pdf ^7^

In this article, the term electronic cigarettes (EC) ^7^ is used to refer to electronic nicotine delivery systems, restricting the analysis to those containing nicotine, and CC is used to denote combustible cigarettes. EC have evolved through generations: from cigalike-type devices (first generation) to vape pens/refillable tanks (second), mods (third), and pods/disposables (fourth) (figure 1). This evolution is not only technological but introduces variations in the ease of refilling and modification, the variety of flavors, and marketing channels—elements relevant to defining regulatory scope and oversight challenges.

## HISTORICAL EVOLUTION OF THE PERUVIAN REGULATORY FRAMEWORK

Since the 1980s, Peru has progressively developed a regulatory framework aimed at tobacco control, with notable strengthening starting in the 1990s.

### Main milestones of the regulatory framework

1991: Law 25357 (1991), which prohibited smoking in enclosed public spaces and constituted a turning point in national tobacco control policy ^8^.

2004: Ratification of the WHO Framework Convention on Tobacco Control (FCTC), which entered into force in 2005 and required the strengthening of regulatory strategies ^9^. In 2006: Law 28705 ^10^ which consolidated prevention and control measures (advertising restrictions, smoke-free environments, health warnings on packaging, and prohibitions on sales to minors).

2008: Regulations of Law 28705 (D.S. 015-2008-SA) ^11^.

2010: Law 29517 ^12^, which reinforced health protection, including 100% smoke-free environments and improved health warnings, with warnings on 50% of the main faces.

 2024: Promulgation of Law 32159, which repealed previous norms and established a comprehensive framework for tobacco, nicotine, and substitute products, explicitly incorporating nicotine delivery systems (ENDS), including nicotine EC ^6^. 

Taken together, this trajectory reflects a progressive shift from a focus primarily centered on combustible tobacco toward one that recognizes a broader ecosystem of nicotine and substitute products, including nicotine EC. This shift is relevant because EC present distinct marketing and use modalities (e.g., refillable or disposable devices, liquids and flavors, digital sales), requiring clear operational definitions and adapted oversight mechanisms to avoid regulatory voids. In this context, it is pertinent to examine how Law 32159 ^6^—the Law for the Control of the Consumption of Tobacco, Nicotine, or Substitute Products for the Protection of Life and Health—addresses these regulatory components and what gaps persist for its effective implementation.

## LAW 32159 APPLIED TO NICOTINE ELECTRONIC CIGARETTES

Law 32159 ^6^ establishes a comprehensive framework for the control of tobacco, nicotine, and substitute products, explicitly incorporating electronic nicotine delivery systems. Its relevant provisions for nicotine EC can be categorized into the following regulatory axes:

100% smoke-free and vape-free environments: The law prohibits smoking or vaping in health and education establishments, public offices, workplaces, enclosed public spaces, and public transport, declaring these environments 100% smoke-free and vape-free (Art. 6).

Mandatory posting of signs in visible places: “smoking and vaping are prohibited in this establishment as they are harmful to health. this environment is one hundred percent smoke-free and vape-free.” (Art. 7).

Advertising, promotion, and sponsorship: Establishes a total ban on advertising, promotion, and sponsorship of tobacco products and their substitutes (Art. 8) and incorporates restrictions for the advertising of nicotine products, including location, content, and mandatory warnings (Art. 9).

Packaging, labeling, and health warnings: Introduces new packaging and labeling standards, providing for health warnings that cover 70% of the main faces on tobacco products, and at least 30% on nicotine products (Art. 10 and 11).

Marketing and access restrictions (protection of minors and supply control): Incorporates limitations on marketing, with an emphasis on sales to minors, the use of flavors attractive to youth, and sales through automatic vending machines (Art. 14).

Oversight and multisectoral governance: Defines multisectoral oversight mechanisms led by the Ministry of Health, local governments, and INDECOPI (Art. 17).

Overall, these axes delineate the current regulatory framework for nicotine EC. Below, the consistency and sufficiency of these provisions are critically examined in light of relevant standards and selected evidence, in order to identify specific gaps and implementation challenges that could limit their effectiveness in practice.

## CRITICAL ANALYSIS BY AXES: SPECIFIC GAPS AND IMPLEMENTATION CHALLENGES

### Flavorings and attractiveness to adolescents

Law 32159 ^6^ incorporates restrictions related to the use of flavors attractive to youth and reinforces control measures in the marketing of nicotine products (Art. 14). This orientation is pertinent in a context where annual electronic cigarette use among schoolchildren stands at relevant levels of 10.8% ^13^, reinforcing the need for preventive measures directed at adolescents.

The main gap lies in operability: if the prohibition/restriction of flavorings is not accompanied by verifiable definitions and criteria, it can be bypassed through nomenclature changes, reformulations, or marketing strategies. Furthermore, effectiveness depends on actual oversight at points of sale and in the informal market, which is significant in a country where it has already been noted that, even with legislation to protect minors and regulate advertising, implementation can be weak; according to the Global Youth Tobacco Survey (GYTS), it reflects alarming accessibility, with 74.6% purchasing combustible cigarettes in a shop. Four out of ten schoolchildren (37.3%) were not refused sales due to their age, with no difference by sex. It was also asked if they saw tobacco use in TV programs, videos, or movies in the last 30 days, and 68.5% of students responded yes. Therefore, stricter controls for the compliance of prohibition laws are recommended ^14^.

Relevant aspects for the regulations:

Operational definition of “attractive flavor” (including descriptors, presentations, and substitutes), 2. Labeling and traceability obligations for the liquid/device, 3. Explicit rules for digital and *delivery* sales, and 4. Inspection and sanction regime applicable to informal sales.

### Packaging, labeling, and health warnings: consistency and sufficiency

Law 32159 ^6^ establishes health warnings of 70% for tobacco products and at least 30% for nicotine products on each of their main faces (Arts. 10-11) ^6^. Although the inclusion of warnings for nicotine products constitutes an advance, the size difference presents a regulatory consistency gap: it could convey an implicit message of lower health relevance or reduce the capacity of warnings to counterbalance the packaging as a promotional vehicle. Experimental studies have shown that warnings covering 75% of the pack are perceived as more effective than those covering 40%, reinforcing that larger warnings communicate risk better and more strongly counteract the promotional effect of the packaging. In this context, the size difference (70% for tobacco vs. 30% for nicotine products) can be interpreted as a regulatory consistency gap and a possible implicit message of lower health relevance for the latter ^15^.

This gap is particularly sensitive in adolescents, considering that standardized surveillance (13-15 years) reports exposure to advertising or promotions at points of sale ^16^. Below, selected evidence focused solely on the adolescent population is presented to contextualize the regulatory relevance of health warnings, without claiming an exhaustive review of health effects. The need for robust warnings can also be supported by recent synthesis evidence reporting the production of a complex toxic mixture leading to endothelial damage through oxidative stress; the use of ENDS is associated with cardiovascular, respiratory, periodontal risks, and other outcomes, with particular concern for adolescents ^5^.

An umbrella review of systematic reviews on electronic nicotine delivery systems found that the use of ENDS is associated with an increased risk of asthma ^17^ and multiple respiratory symptoms (pooled AOR 1.39 for asthma and 1.49 for respiratory symptoms), as well as asthma exacerbation in adolescents and adults. The same work identified limited but suggestive evidence of cardiovascular risk, with increases in blood pressure and heart rate, oxidative stress, endothelial dysfunction, and platelet activation, and only very limited and inconclusive evidence regarding cancer ^17^. Adolescent ENDS users present between 31% and 46% higher risk of asthma and other chronic respiratory symptoms (OR 1.31-1.46) and around 49% higher risk of COPD or compatible symptoms (OR 1.49; 95% CI = 1.36-1.65) ^5^.

In a longitudinal study of adolescents in Texas, 32.9% of the weighted sample initiated ENDS use at some point between 2014 and 2018 (1324 out of 461,069). Among those who used ENDS, 67.6% ended up initiating some combustible tobacco product (cigarettes, cigars, or hookah), while only 32.4% did not. Within this group of ENDS users, 39.1% initiated ENDS and combustible tobacco in the same wave (dual initiation), 19.4% began using combustible tobacco in a wave subsequent to ENDS initiation, and 9.1% had initiated combustible tobacco before ENDS, showing that approximately two out of three adolescents who tried ENDS made some transition toward the use of combustible products ^18^.

Relevant aspects for the regulations:

Content of specific warnings for nicotine (dependence, risk in adolescents/non-users), 2. Technical criteria for legibility, location, and rotation, 3. Requirements for imported products and those marketed in informal environments.

### Advertising, promotion, sponsorship, and digital marketing

Among adolescents, there are signs of exposure to advertising/promotions at points of sale ^16^, which requires that the regulations establish operational definitions and procedures that allow for clear distinctions between: display, promotions, discounts, “merchandising,” undercover sponsorship, and digital advertising (including third-party content).

Relevant aspects for the regulations:

Verification and sanction mechanisms, including digital commerce, prohibiting advertising, promotion, and sponsorship of tobacco products and their substitutes (Art. 8) and incorporating restrictions for the advertising of nicotine products (Art. 9). The central challenge is that efficacy depends on how direct and indirect advertising is defined and controlled, especially in environments where exposure occurs at points of sale and potentially in digital media. 2. Point-of-sale display rules (visibility, promotional material), 3. Monitoring and reporting procedures, and 4. Sanction routes and inter-institutional coordination.

### Marketing and access control

Law 32159 ^6^ incorporates limitations on marketing and reinforces the protection of minors (Art. 14). However, a frequent gap between norm and practice is compliance: even when restrictions exist to protect minors, implementation may be insufficient if not accompanied by effective verification, inspections, and sanctions ^14^. Surveillance evidence (although referring to combustible cigarettes) shows that among those who tried to buy cigarettes, a significant proportion was not prevented by their age ^16^, illustrating the need for control mechanisms applicable also to nicotine EC.

Relevant aspects for the regulations:

Age verification protocols (including *delivery* and digital commerce), 2. Inspections and graduated sanctions for points of sale, 3. Criteria for seizure/retention of products without labeling or from informal origins, and 4. Operational articulation with local governments and competent authorities.

### Multisectoral oversight and informal market

The law provides for multisectoral oversight led by the Ministry of Health, local governments, and INDECOPI (Art. 17). The key gap is that if oversight lacks operational capacity, coordination, and homogeneous criteria, a substantial part of the market may shift to or remain in informal and digital circuits, weakening measures on flavors, warnings, and access control.

This risk is consistent with previous warnings that the implementation of provisions on advertising and access to minors can be weak, making it essential to design monitoring and control mechanisms ^14^. In nicotine EC, the problem is aggravated by the diversity of devices, nicotine presentations, and available liquids with flavorings that also produce adverse events ^19^^-^^21^, as well as by sales channels with less traceability.

Relevant aspects for the regulations:

Clear distribution of competencies by type of infraction, 2. Unified inspection and evidence protocols (labeling, advertising, sales to minors), 3. Coordination with municipalities and control of digital commerce, and 4. Simple compliance goals and indicators.

### Specific taxes and price policy

Although Law 32159 ^6^ consolidates regulatory measures on environments, advertising, warnings, and marketing, comprehensive control usually also requires price policy instruments. In line with the WHO MPOWER package, which identifies raising taxes (Raise taxes on tobacco) as the most effective measure to reduce tobacco consumption ^22^. Article 6 of the WHO Framework Convention on Tobacco Control establishes that States Parties should implement price and tax policies to reduce the demand for tobacco products through specific or mixed systems and regular adjustments ^23^. International guidelines recommend incorporating robust fiscal components as part of a comprehensive control strategy ^24^. In a scenario where annual electronic cigarette use among schoolchildren reaches a relevant level of 10.8% ^13^, the absence of specific fiscal measures may maintain high economic accessibility and limit the deterrent capacity of the regulatory package. Considering that nicotine products are not taxed with the Selective Consumption Tax despite generating negative externalities similar to tobacco products, the question to answer would be: Is it necessary to incorporate specific taxes for nicotine EC as a complementary measure, ideally articulated with informal market control and oversight mechanisms?.

## PRIORITY GAPS AND IMPLEMENTATION CHALLENGES

Overall, the analysis by axes suggests that the effectiveness of Law 32159 ^6^ for nicotine EC will depend largely on its regulatory operationalization and oversight capacity ^25^. Gaps in definition and verifiability persist regarding flavors attractive to adolescents and their possible evasion through commercial presentations or marketing strategies, particularly relevant in a context where electronic cigarette use among schoolchildren reaches significant levels of 10.8% ^13^ and where it has been warned that the implementation of protective provisions may be weak without effective control mechanisms ^14^. Likewise, gaps in communicational consistency and sufficiency in health warnings are identified, considering that commercial exposure at points of sale has been documented in adolescents ^16^, reinforcing the need for technical standards and homogeneous verification procedures. In parallel, the control of advertising and promotion requires operational precision, especially to address indirect modalities and digital environments; and multisectoral oversight faces the challenge of coordinating real competencies and capacities to prevent the informal market from weakening restrictions on flavors, warnings, and access for minors; SUNAT seized more than 8 million smuggled cigarettes declared as *tissue* paper ^26^. Forty-four vape providers were inspected, and it was found that none fully complied with Spanish labeling and regulatory requirements ^27^. Finally, a public policy void is observed regarding price instruments and specific taxes, relevant for reducing accessibility in adolescents ^13^, the evaluation of which could be integrated into a more complete regulatory strategy. In this framework, the pending regulations constitute a critical component for translating the legal text into implementable and enforceable measures ^25^.

## CONCLUSIONS

Law 32159 constitutes an advance by explicitly incorporating nicotine electronic cigarettes within a comprehensive framework for the control of tobacco, nicotine, and substitute products. However, its effectiveness will fundamentally depend on the precision and operability of its regulations and on oversight capacity.

Provisions related to the protection of adolescents, particularly those concerning attractive flavors, marketing, and advertising, require verifiable operational definitions and control procedures that reduce evasion through commercial strategies, digital sales, and informal channels.

The difference in the standard for health warnings between tobacco products and nicotine products presents a challenge for regulatory consistency and risk communication. It is a priority that the regulations establish clear technical criteria ensuring effective, enforceable warnings applicable to products imported and marketed outside formal circuits.

The actual implementation of the law faces structural challenges: multisectoral coordination, inspection and sanction capacities, and control of the informal market. Without addressing these components, there is a risk that substantive restrictions (flavors, warnings, access for minors) will be neutralized in practice.

Finally, to consolidate a comprehensive response, it is pertinent to evaluate the incorporation of complementary public policy instruments, including fiscal and price measures in coordination with oversight strategies, in order to reduce accessibility and strengthen the effectiveness of the regulatory package.
