# Supplementary material for: Nicotine e-cigarettes in Peru: analysis of law 32159 against international evidence and gaps for adolescent protection
Source: Rev Peru Med Exp Salud Publica. 2026 Mar 31;43(1):119–25. doi: 10.17843/rpmesp.2026.431.15979 (PMC13245993; doi:10.17843/rpmesp.2026.431.15979)
Supplement: Translation

# Translation: Cigarrillos electrónicos con nicotina en Perú: análisis de la ley 32159 frente a la evidencia internacional y brechas para la protección adolescente

**Authors:** Jesus Chirinos-Caceres, Eva Chanamé-Ampuero, Abel Limache-García, Alfonso Zavaleta-Martínez-Vargas

**Affiliations:** 1 Departamento Académico de Salud Pública, Administración y Ciencias Sociales, Facultad de Salud Pública y Administración, Universidad Peruana Cayetano Heredia, Lima, Perú.; 2 Centro de información y educación para la prevención del abuso de drogas (CEDRO), Lima, Perú.; 3 Departamento Académico de Enfermería, Facultad de Enfermería, Universidad Peruana Cayetano Heredia, Lima, Perú.; 4 Departamento Académico de Ciencias Celulares y Moleculares, Facultad de Ciencias e Ingeniería, Universidad Peruana Cayetano Heredia, Lima, Perú.; 5 Facultad de Medicina, Universidad San Ignacio de Loyola, Lima, Perú.

**Keywords:** Cigarrillos electrónicos, Sistemas Electrónicos de Administración de Nicotina, Salud Pública, Prevención del Hábito de Fumar, Control del Tabaco, Perú

## Abstract

El artículo analiza la regulación de los cigarrillos electrónicos (CE) con nicotina en el Perú, con énfasis en la Ley 32159 y su capacidad para proteger la salud de los adolescentes a la luz de la evidencia internacional. Aunque suelen percibirse como menos dañinos que los cigarrillos combustibles, estudios vinculan su uso con riesgos cardiovasculares, respiratorios, además de mayor probabilidad de transición hacia el consumo de tabaco combustible en adolescentes. Se revisa la evolución del marco normativo peruano de control del tabaco y se reconoce que la Ley 32159 constituye un avance al incorporar explícitamente los sistemas electrónicos de administración de nicotina dentro de un enfoque regulatorio integral. En ese sentido, la norma aborda ambientes 100% libres de humo y vapeo, prohibición de publicidad, promoción y patrocinio, advertencias sanitarias, restricciones de comercialización, fiscalización multisectorial e incorpora el Art. 5.3 del Convenio Marco para el Control del Tabaco referida a la interferencia de la industria en las políticas de control del consumo de productos de tabaco, nicotina y sucedáneos de ambos. No obstante, persisten brechas entre la norma y su implementación: definiciones insuficientes sobre saborizantes, exigencias menores de advertencias sanitarias y limitada capacidad para controlar publicidad indirecta y digital, en un contexto de mercado informal y comercio en línea. La ausencia de medidas fiscales debilita la política pública. En conclusión, el impacto de la ley dependerá de un reglamento preciso, fiscalización efectiva e instrumentos complementarios como políticas de precio e impuestos.

## INTRODUCCIÓN

En el 2018, las Academias Nacionales de Ciencias, Ingeniería y Medicina (NASEM, por sus siglas en inglés) publicaron las consecuencias del uso de cigarrillos electrónicos (CE) en la salud pública ^1^. Antes, el 10 de mayo de 2016, la Administración de Alimentos y Medicamentos (FDA, por sus siglas en inglés) emitió una regla ^2^ para extender la regulación a todos los productos de tabaco, incluyendo cigarrillos electrónicos ya que cumplen con la definición legal de un producto de tabaco.

Este “Reglamento de consideración” ^2^ permite a la FDA regular la fabricación, distribución y mercadeo de productos de tabaco como los e-cigarrillos e incluye provisiones automáticas tales como restricciones en la venta a los jóvenes. Aunque varias formas de nicotina electrónica ^3^^)^ provistas por baterías han existido durante más de una década, su popularidad, especialmente entre los jóvenes, ha aumentado en los últimos cinco años ^4^. A diferencia de los cigarrillos convencionales (CC), los cigarrillos electrónicos no se queman y no contienen la mayor parte de los 7000 constituyentes químicos estimados, presentes en el humo del tabaco. Por lo tanto, se cree que los cigarrillos electrónicos son más seguros que los CC; sin embargo, sí producen exposiciones a la nicotina y a una variedad de otros componentes potencialmente dañinos ^1^. Evidencia reciente basada en una revisión paraguas que integró 69 revisiones sistemáticas, sugiere que el uso de sistemas electrónicos de administración de nicotina se asocia con mayores riesgos cardiovasculares y respiratorios, así como con problemas de salud mental y abuso de sustancias, con especial preocupación en adolescentes. Entre los hallazgos reportados se describen incrementos en frecuencia cardiaca y presión arterial, además de disminución de la función pulmonar y mayor probabilidad de asma en usuarios ^5^. El daño también ocurre si los jóvenes comienzan su uso de tabaco con cigarrillos electrónicos y luego hacen la transición a los de combustión, o si los fumadores adultos de los CC agregan los e-cigarrillos para complementar su hábito de fumar, en lugar de dejar los cigarrillos de tabaco combustible completamente ^1^.

El objetivo de este artículo es realizar un análisis crítico de la normativa peruana aplicable a los CE con nicotina, incluida su evolución histórica y la Ley 32159 ^6^, contrastando sus disposiciones regulatorias con evidencia seleccionada y estándares relevantes, para identificar brechas específicas y desafíos de implementación, sin desarrollar una revisión amplia de sus efectos en salud.

## ALCANCE

En este artículo, el término cigarrillos electrónicos (CE) ^7^ se emplea para referirse a los sistemas electrónicos de administración de nicotina, restringiendo el análisis a aquellos que contienen nicotina, y se utiliza CC para denominar a los cigarrillos combustibles. Los CE han evolucionado por generaciones: desde dispositivos tipo *cigalikes* (primera generación) pasando por plumas de vapeo/tanques recargables (segunda), mods (tercera) y pods/desechables (cuarta) (figura 1). Esta evolución no es solo tecnológica, sino que introduce variaciones en la facilidad de recarga y modificación, la oferta de sabores y los canales de comercialización, elementos relevantes para definir el alcance regulatorio y los retos de fiscalización.

**Figura 1:** Los sistemas electrónicos de suministro de nicotina (ENDS) se clasifican según generación.

Fuente: CDC. (2019) e-cigarette, or vaping, products and dictionary

## EVOLUCIÓN HISTÓRICA DEL MARCO REGULATORIO PERUANO

Desde la década de 1980, el Perú ha desarrollado progresivamente un marco regulatorio orientado al control del consumo de tabaco, con un fortalecimiento notable a partir de los años noventa.

### Hitos principales del marco normativo

1991: Ley 25357 (1991), que prohibió fumar en espacios cerrados de uso público y constituyó un punto de inflexión en la política nacional de control del tabaco ^8^.

2004: Ratificación del Convenio Marco de la OMS para el Control del Tabaco (CMCT), cuyo ingreso en vigor en 2005 obligó a robustecer las estrategias regulatorias ^9^.

En 2006: Ley 28705 ^10^ que consolidó medidas de prevención y control (restricciones a la publicidad, ambientes libres de humo, advertencias sanitarias en empaques y prohibiciones de venta a menores).

2008: Reglamento de la Ley 28705 (D.S. 015-2008-SA) ^11^.

2010: Ley 29517 ^12^, que reforzó la protección sanitaria, incluyendo ambientes 100 % libres de humo y mejora las advertencias sanitarias, advertencias en el 50% de las caras principales.

2024: Promulgación de la Ley 32159, que derogó las normas e instauró un marco integral para productos de tabaco, nicotina y sus sucedáneos, incorporando explícitamente los sistemas de administración de nicotina (SEAN), incluidos los CE con nicotina ^6^.

En conjunto, esta trayectoria refleja un desplazamiento progresivo desde un enfoque centrado principalmente en el tabaco combustible hacia uno que reconoce un ecosistema más amplio de productos de nicotina y sucedáneos, incluidos los CE con nicotina. Este cambio es relevante porque los CE presentan modalidades de comercialización y uso distintas (p. ej., dispositivos recargables o desechables, líquidos y sabores, venta digital), lo que exige definiciones operativas claras y mecanismos de fiscalización adaptados para evitar vacíos normativos. En ese contexto, resulta pertinente examinar cómo la Ley 32159 ^6^ - Ley del Control del Consumo de Productos de Tabaco, Nicotina o Sucedáneos de Ambos para la Protección de la Vida y la Salud aborda estos componentes regulatorios y qué brechas persisten para su implementación efectiva.

## LEY 32159 APLICADA A CIGARRILLOS ELECTRÓNICOS CON NICOTINA

La Ley 32159 ^6^, establece un marco integral de control de productos de tabaco, nicotina y sus sucedáneos, incorporando explícitamente los sistemas electrónicos de administración de nicotina. Sus disposiciones relevantes para los CE con nicotina podemos mencionar los siguientes ejes regulatorios:

Ambientes 100% libres de humo y emisiones de vapeo: La ley prohíbe fumar o vapear en establecimientos de salud y educación, dependencias públicas, lugares de trabajo, espacios públicos cerrados y transporte público, declarando estos ambientes como 100 % libres de humo y emisiones de vapeo (Art. 6).

Obliga colocar anuncio en lugares visibles: “está prohibido fumar y vapear en este establecimiento por ser dañino para la salud. este ambiente es cien por ciento libre de humo y emisiones de vapeo”. (Art. 7).

Publicidad, promoción y patrocinio: Establece la prohibición total de publicidad, promoción y patrocinio de productos de tabaco y sus sucedáneos (Art. 8) e incorpora restricciones para la publicidad de productos de nicotina, incluyendo ubicación, contenido y advertencias obligatorias (Art. 9).

Empaquetado, etiquetado y advertencias sanitarias: Introduce nuevos estándares de empaquetado y etiquetado, disponiendo advertencias sanitarias que cubren el 70 % de las caras principales en productos de tabaco, y al menos el 30 % en productos de nicotina (Art. 10 y 11).

Comercialización y restricciones de acceso (protección de menores y control de oferta): Incorpora limitaciones a la comercialización, con énfasis en la venta a menores, uso de saborizantes atractivos para jóvenes y expendio mediante máquinas automáticas (Art. 14).

Fiscalización y gobernanza multisectorial: Define mecanismos de fiscalización multisectorial a cargo del Ministerio de Salud, gobiernos locales e INDECOPI (Art. 17).

En conjunto, estos ejes delimitan el marco regulatorio vigente para los CE con nicotina. A continuación, se examina críticamente la coherencia y suficiencia de estas disposiciones a la luz de estándares relevantes y evidencia seleccionada, con el fin de identificar brechas específicas y desafíos de implementación que podrían limitar su efectividad en la práctica.

## ANÁLISIS CRÍTICO POR EJES: BRECHAS ESPECÍFICAS Y DESAFÍOS DE IMPLEMENTACIÓN

### Saborizantes y atractivo para adolescentes

La Ley 32159 ^6^ incorpora restricciones vinculadas al uso de saborizantes atractivos para jóvenes y refuerza medidas de control en la comercialización de productos de nicotina (Art. 14). Esta orientación resulta pertinente en un contexto donde el consumo anual de cigarrillo electrónico en escolares se ubica en niveles relevantes del 10,8% ^13^, lo que refuerza la necesidad de medidas preventivas dirigidas a adolescentes.

La brecha principal está en la operatividad: si la prohibición/restricción de saborizantes no se acompaña de definiciones y criterios verificables, puede ser eludida mediante cambios de nomenclatura, reformulaciones o estrategias de mercadeo. Además, la efectividad depende de su fiscalización real en puntos de venta, y mercado informal, lo que no es menor en un país donde ya se ha advertido que, aun existiendo legislación para proteger a menores y regular publicidad, su implementación puede ser débil; según la encuesta mundial de consumo de tabaco en adolescentes (EMTA), refleja una alarmante accesibilidad, que el 74,6% compraban cigarrillos combustibles en una tienda. A cuatro de diez escolares (37,3%) no se le negó la venta debido a su edad, sin diferencia según sexo. Se consultó también si vieron consumir tabaco en programas de TV, videos o películas de cine, en los últimos 30 días, el 68,5% de estudiantes respondió que sí. Por eso, recomiendan controles más estrictos para el cumplimiento de las leyes de prohibición ^14^.

Aspectos relevantes para el reglamento:

1. Definición operativa de “saborizante atractivo” (incluyendo descriptores, presentaciones y sustitutos), 2. Obligaciones de rotulado y trazabilidad del líquido/dispositivo, 3. Reglas explícitas para venta digital y *delivery*, y 4. Régimen de inspección y sanción aplicable a ventas informales.

### Empaquetado, etiquetado y advertencias sanitarias: coherencia y suficiencia

La Ley 32159 ^6^^)^ establece advertencias sanitarias del 70% para productos de tabaco y de al menos 30% para productos de nicotina de cada una de sus caras principales (Arts. 10-11) ^6^. Aunque la inclusión de advertencias para productos de nicotina constituye un avance, la diferencia de tamaño plantea una brecha de coherencia regulatoria: podría transmitir un mensaje implícito de menor relevancia sanitaria o reducir la capacidad de las advertencias para contrapesar el empaque como vehículo promocional. Estudios experimentales han mostrado que advertencias que cubren el 75% del paquete son percibidas como más efectivas que aquellas que cubren el 40%, reforzando que las advertencias de mayor tamaño comunican mejor el riesgo y contrapesan con mayor fuerza el efecto promocional del empaque. En este contexto, la diferencia de tamaño (70% para tabaco frente a 30% para productos de nicotina) puede interpretarse como una brecha de coherencia regulatoria y un posible mensaje implícito de menor relevancia sanitaria para los segundos ^15^.

Esta brecha es particularmente sensible en adolescentes, considerando que la vigilancia estandarizada (13-15 años) reporta exposición a publicidad o promociones en puntos de venta ^16^. A continuación, se presenta evidencia seleccionada centrada en población adolescente únicamente para contextualizar la relevancia regulatoria de las advertencias sanitarias, sin pretender una revisión exhaustiva de efectos en salud. La necesidad de advertencias robustas también puede sustentarse en evidencia reciente de síntesis que reporta la producción de una mezcla tóxica compleja que lleva a daño endotelial por estrés oxidativo en el uso de ENDS se asocia con riesgos cardiovasculares, respiratorios, periodontales y otros desenlaces, con especial preocupación en los adolescentes ^5^.

Una revisión paraguas de revisiones sistemáticas sobre sistemas electrónicos de administración de nicotina encontró que el uso de ENDS se asocia con un mayor riesgo de asma ^17^ y múltiples síntomas respiratorios (AOR agrupado 1,39 para asma y 1,49 para síntomas respiratorios), así como con exacerbación del asma en adolescentes y adultos. El mismo trabajo identificó evidencia limitada pero sugestiva de riesgo cardiovascular, con aumentos de presión arterial y frecuencia cardíaca, estrés oxidativo, disfunción endotelial y activación plaquetaria, y solo evidencia muy limitada y no concluyente en relación con cáncer ^17^. Adolescentes usuarios de ENDS presentan entre 31% y 46% más riesgo de asma y otros síntomas respiratorios crónicos (OR 1,31-1,46) y alrededor de 49% más riesgo de EPOC o síntomas compatibles (OR 1,49; IC 95 %= 1,36-1,65) ^5^.

En un estudio longitudinal de adolescentes en Texas, el 32,9% de la muestra ponderada inició el uso de ENDS alguna vez entre 2014 y 2018 (1324 de 461 069). Entre quienes usaron ENDS, el 67,6% terminó iniciando algún producto de tabaco combustible (cigarrillos, puros o narguile), mientras que solo el 32,4% no lo hizo. Dentro de este grupo de usuarios de ENDS, el 39,1% inició ENDS y tabaco combustible en la misma ola (inicio dual), el 19,4% comenzó a usar tabaco combustible en una ola posterior al inicio de ENDS y el 9,1% había iniciado tabaco combustible antes de ENDS, lo que muestra que aproximadamente dos de cada tres adolescentes que probaron ENDS realizaron alguna transición hacia el uso de productos combustibles ^18^.

Aspectos relevantes para el reglamento:

1. Contenido de advertencias específicas para nicotina (dependencia, riesgo en adolescentes/no usuarios), 2. Criterios técnicos de legibilidad, ubicación y rotación, 3. Exigencias para productos importados y comercializados en entornos no formales. 

### Publicidad, promoción, patrocinio y marketing digital

En adolescentes, existe señal de exposición a publicidad/promociones en puntos de venta ^16^, lo que exige que el reglamento aterrice definiciones operativas y procedimientos que permitan distinguir con claridad: exhibición, promociones, descuentos, “merchandising”, patrocinio encubierto y publicidad digital (incluidos contenidos de terceros).

Aspectos relevantes para el reglamento:

1. Mecanismos de verificación y sanción, incluyendo comercio digital, prohibir la publicidad, promoción y patrocinio de productos de tabaco y sus sucedáneos (Art. 8) e incorpora restricciones para publicidad de productos de nicotina (Art. 9). El desafío central es que la eficacia depende de cómo se defina y controle la publicidad directa e indirecta, especialmente en entornos donde la exposición ocurre en puntos de venta y potencialmente en medios digitales. 2. Reglas de exhibición en punto de venta (visibilidad, material promocional), 3. Procedimientos de monitoreo y reporte, y 4. Rutas de sanción y coordinación interinstitucional.

### Comercialización y control de acceso

La Ley 32159 ^6^ incorpora limitaciones a la comercialización y refuerza la protección de menores (Art. 14). Sin embargo, una brecha frecuente entre norma y práctica es el cumplimiento: aun cuando existen restricciones para proteger a menores, la implementación puede ser insuficiente si no se acompaña de verificación efectiva, inspecciones y sanciones ^14^. La evidencia de vigilancia (aunque referida a cigarrillos combustibles) muestra que, entre quienes intentaron comprar cigarrillos, una proporción importante no fue impedida por su edad ^16^, lo que ilustra la necesidad de mecanismos de control aplicables también a CE con nicotina.

Aspectos relevantes para el reglamento:

1. Protocolos de verificación de edad (incluyendo *delivery* y comercio digital), 2. Inspecciones y sanciones escalonadas para puntos de venta, 3. Criterios de decomiso/retención para productos sin rotulado o de origen informal, y 4. Articulación operativa con gobiernos locales e instancias competentes.

### Fiscalización multisectorial y mercado informal

La ley dispone fiscalización multisectorial a cargo del Ministerio de Salud, gobiernos locales e INDECOPI (Art. 17). La brecha clave es que, si la fiscalización no cuenta con capacidades operativas, coordinación y criterios homogéneos, una parte sustancial del mercado puede desplazarse o permanecer en circuitos informales y digitales, debilitando medidas sobre sabores, advertencias y control de acceso.

Este riesgo es coherente con advertencias previas de que la implementación de disposiciones sobre publicidad y acceso a menores puede ser débil, lo que hace imprescindible diseñar mecanismos de seguimiento y control ^14^. En CE con nicotina, la problemática se agrava por la diversidad de dispositivos, presentación de la nicotina y líquidos disponibles con saborizantes que también producen eventos adversos ^19^^-^^21^, así como por canales de venta con menor trazabilidad.

Aspectos relevantes para el reglamento:

1. Distribución clara de competencias por tipo de infracción, 2. Protocolos unificados de inspección y evidencia (rotulado, publicidad, venta a menores), 3. Coordinación con municipalidades y control de comercio digital, y 4. Metas e indicadores simples de cumplimiento.

### Impuestos específicos y política de precio

Aunque la Ley 32159 ^6^^)^ consolida medidas regulatorias sobre ambientes, publicidad, advertencias y comercialización, el control integral suele requerir también instrumentos de política de precio. En línea con el paquete MPOWER de la OMS, que identifica el aumento de impuestos (Raise taxes on tobacco) como la medida más efectiva para reducir el consumo de tabaco ^22^. El Artículo 6 del Convenio Marco de la OMS para el Control del Tabaco, establece que los Estados Parte deberían aplicar políticas de precio e impuestos para reducir la demanda de productos de tabaco mediante sistemas específicos o mixtos y ajustes regulares ^23^. Los lineamientos internacionales recomiendan incorporar componentes fiscales robustos como parte de una estrategia integral de control ^24^. En un escenario donde el consumo anual de cigarrillo electrónico en escolares alcanza un nivel relevante de 10,8% ^13^, la ausencia de medidas fiscales específicas puede mantener una alta accesibilidad económica y limitar la capacidad disuasiva del paquete regulatorio.

Considerando que los productos de nicotina no están gravados con el impuesto Selectivo al Consumo a pesar de generar externalidades negativas a los productos de tabaco, la pregunta a responder sería: ¿Es necesario incorporar impuestos específicos para CE con nicotina como medida complementaria?, idealmente articulada con control de mercado informal y mecanismos de fiscalización.

## BRECHAS PRIORITARIAS Y DESAFÍOS DE IMPLEMENTACIÓN

En conjunto, el análisis por ejes sugiere que la efectividad de la Ley 32159 ^6^ para los CE con nicotina dependerá en gran medida de su operativización reglamentaria y de la capacidad de fiscalización ^25^. Persisten brechas de definición y verificabilidad en torno a saborizantes atractivos para adolescentes y su posible elusión mediante presentaciones comerciales o estrategias de mercadeo, particularmente relevante en un contexto donde el consumo de cigarrillo electrónico en escolares alcanza niveles importantes 10,8% ^13^ y donde se ha advertido que la implementación de disposiciones protectoras puede ser débil si no se acompaña de mecanismos efectivos de control ^14^. Asimismo, se identifican brechas de coherencia comunicacional y suficiencia en las advertencias sanitarias, considerando que la exposición comercial en puntos de venta ha sido documentada en adolescentes ^16^, lo que refuerza la necesidad de estándares técnicos y procedimientos de verificación homogéneos. En paralelo, el control de la publicidad y promoción requiere precisiones operativas, especialmente para abordar modalidades indirectas y entornos digitales; y la fiscalización multisectorial enfrenta el reto de coordinar competencias y capacidades reales para evitar que el mercado informal debilite restricciones sobre sabores, advertencias y acceso a menores; SUNAT incautó más de 8 millones de cigarrillos de contrabando declarados como papel *tissue*^26^. Se inspeccionaron 44 proveedores de vapeadores y se encontró que ninguno cumplía totalmente con el etiquetado en español y con las exigencias de la normativa ^(^^27^. Finalmente, se observa un vacío de política pública respecto a instrumentos de precio e impuestos específicos, relevantes para reducir la accesibilidad en adolescentes ^13^, cuya evaluación podría integrarse a una estrategia regulatoria más completa. En este marco, el reglamento pendiente constituye un componente crítico para traducir el texto legal en medidas implementables y fiscalizables ^25^.

## CONCLUSIONES

La Ley 32159 constituye un avance al incorporar explícitamente los cigarrillos electrónicos con nicotina dentro de un marco integral de control de productos de tabaco, nicotina y sucedáneos. Sin embargo, su efectividad dependerá fundamentalmente de la precisión y operatividad de su reglamentación y de la capacidad de fiscalización.

Las disposiciones vinculadas a la protección de adolescentes en particular las relacionadas con saborizantes atractivos, comercialización y publicidad, requieren definiciones operativas verificables y procedimientos de control que reduzcan la elusión por estrategias comerciales, venta digital y canales informales.

La diferencia en el estándar de advertencias sanitarias entre productos de tabaco y productos de nicotina plantea un reto de coherencia regulatoria y de comunicación de riesgo. Es prioritario que el reglamento establezca criterios técnicos claros que aseguren advertencias efectivas, fiscalizables y aplicables a productos importados y comercializados fuera del circuito formal.

La implementación real de la ley enfrenta desafíos estructurales: coordinación multisectorial, capacidades de inspección y sanción, y control del mercado informal. Sin abordar estos componentes, existe riesgo de que las restricciones sustantivas (sabores, advertencias, acceso de menores) se neutralicen en la práctica.

Finalmente, para consolidar una respuesta integral, resulta pertinente evaluar la incorporación de instrumentos complementarios de política pública, incluyendo medidas fiscales y de precio en articulación con estrategias de fiscalización, a fin de reducir la accesibilidad y fortalecer la efectividad del paquete regulatorio.
